# Requirement Assessment of the Relative Spatial Accuracy of a Motion-Constrained GNSS/INS in Shortwave Track Irregularity Measurement

**DOI:** 10.3390/s19235296

**Published:** 2019-12-01

**Authors:** Quan Zhang, Qijin Chen, Xiaoji Niu, Chuang Shi

**Affiliations:** 1 GNSS Research Centre, Wuhan University, Wuhan 430079, China; zhangquan@whu.edu.cn (Q.Z.); xjniu@whu.edu.cn (X.N.); 2 Collaborative Innovation Centre of Geospatial Technology, Wuhan University, Wuhan 430079, China; 3 School of Electronic and Information Engineering, Beihang University, Beijing 100000, China; shichuang@buaa.edu.cn

**Keywords:** spatial relative accuracy, track irregularity, Allan variance, GNSS/INS, motion constraints

## Abstract

Modern railway track health monitoring requires high accuracy measurements to ensure comfort and safety. Although Global Navigation Satellite System/Inertial Navigation System (GNSS/INS) integration has been extended to track geometry measurements to improve the work efficiency, it has been questioned due to its positioning accuracy at the centimeter or millimeter level. We propose the relative spatial accuracy based on the accuracy requirement of track health monitoring. A requirement assessment of the spatial relative accuracy is conducted for shortwave track irregularity measurements based on evaluation indicators and relative accuracy calculations. The threshold values of the relative spatial accuracy that satisfy the constraints of shortwave track irregularity measurements are derived. Motion-constrained GNSS/INS integration is performed to improve the navigation accuracy considering the dynamic characteristics of the track geometry measurement trolley. The results of field tests show that the mean square error and the Allan deviation of the relative position errors of motion-constrained GNSS/INS integration are smaller than 0.67 mm and 0.16 mm, respectively, which indicates that this approach meets the accuracy requirements of shortwave track irregularities, especially vertical irregularities. This work can provide support for the application of GNSS/INS systems in track irregularity measurement.

## 1. Introduction

Modern railways require high-accuracy track measurements for health monitoring because passenger safety and travel comfort or smoothness largely depends on accurate tracks, especially for high-speed railways, and tiny track irregularities (i.e., track deformation) can generate a force large enough to affect the safety and speed of transportation [1,2,3]. Hence, track geometry measurements are of critical importance for the maintenance and adjustment of tracks. There are different accuracy requirements concerning the track course smoothness (quantified by the relative accuracy or inner accuracy) and the absolute position of the track in the reference frame (indicating the absolute accuracy or outer accuracy) [4,5]. Relative accuracy must be guaranteed because track irregularities result in lateral accelerations that must be taken into account in addition to nominal accelerations to ensure safety [1,5]. There are several types of frequencies that can be dangerous for trains and infrastructure; shortwave effects can influence coaches and bridges, and the entire train composition can be affected by longwave effects [2,5].

Most research on the application of Global Navigation Satellite System/Inertial Navigation System (GNSS/INS) systems in track geometry measurements focuses on the absolute accuracy of integrated navigation [1,6,7], representing the total navigation error relative to zero or the mean value [1,8], and the long-term (e.g., more than 10 s) systematic error is dominant [9]. The corresponding common evaluation methods are statistical approaches, e.g., the circular error probable (CEP), the spherical error probable (SEP), and root mean square (RMS) [9,10,11]. These statistics can only reflect the level of overall variation and cannot show the detailed relative variation in navigation errors on different time scales. It has always been questionable why the GNSS/INS systems of centimeter-level positioning can achieve millimeter precision measurement. In fact, this kind of precision measurement is essentially a spatial relative measurement, and it is more concerned with the relative variation of the navigation error on different spatial scales, not the overall error [1,12]. Chen et al. [12] proposed the method of measuring railway track irregularities based on the three-dimensional positions provided by a GNSS/INS integrated system; this approach takes advantage of the relative measurements of the GNSS/INS and is capable of measuring track irregularities with a 1 mm relative accuracy. Mostafa et al. [13] noted that the relative accuracy of the position and orientation system (POS) is influenced by the high-frequency sample-to-sample error of the orientation accuracy of the smoothed navigation solution and that the relative accuracy of the orientation is a function of the gyro noise and residual bias after smoothing. The authors of this work assessed the importance of the relative accuracy at different time scales with the GNSS/INS system in POS applications for mobile mapping and surveying; additionally, the Allan variance, representing the RMS random error as a function of the average time, was presented as an evaluation method of the GNSS/INS relative accuracy [14]. Although some studies have demonstrated the importance of the GNSS/INS relative measurement accuracy in track geometry measurement, the relationship between the GNSS/INS relative accuracy and track irregularity has not been further analyzed; therefore, there is no theoretical support for the application of GNSS/INS integrated navigation in track irregularity measurement.

There are many studies on the track irregularity measuring based on the use of some topographic devices (e.g., total station) on a trolley and bogie-mounted sensors (e.g., accelerometer and gyroscopes) on a dedicated track recording vehicle to measure the accurate track geometry [15,16,17], but these kinds of measurements are either inefficient due to the free stationing of the total station or of limited accuracy because of high speed or sensor performance. With the development of inertial sensors and GNSS/INS systems, the positioning system has been increasingly applied in the lightweight track geometry surveying trolleys for high-accuracy track measurements [12,18,19]. Inertial sensors (typically including gyroscopes and accelerometers) are often mounted on the axle box or bogie of an in-service vehicle to monitor the track irregularity through the lateral and vertical motion derived from an axle box-mounted accelerometer or a bogie-mounted yaw rate gyro [20,21,22]. However, an integral divergence issue can occur over time due to the sensor errors of the gyroscopes and accelerometers, and this error cannot be well restrained by high-pass or low-pass filtering. To overcome the integral divergence over time, multi-sensor fusion methods have been applied in railway track measurements. Jiang et al. [23] utilized the integration of the inertial sensor combined with a zero-velocity updating technique and a sub-decimeter scale landmark to obtain an absolute accuracy of 1 mm. Li et al. [24] presented a laser-aided INS/odometer integration method for subway applications based on the position updates provided by the laser scanner. Dong et al. [25] designed an algorithm and instrument for rapid detection of rail surface defects and vertical short-wave irregularities based on the fiber optic gyro (FOG) and odometer.

The combination of the Global Navigation Satellite System (GNSS) and Inertial Navigation System (INS) has been increasingly applied in track measurement solutions. The GNSS/INS solution utilizes GNSS measurements to obtain the INS solution and provide an integrated navigation solution that retains the INS dynamic accuracy but has the absolute accuracy of GNSS [26]. Lück et al. [6] measured the line characteristics and long-wavelength irregularities of tracks with up to a 150-m wavelength using a differential Global Positioning System (DGPS) and INS mounted on dedicated track inspection trains, and they clearly observed a millimeter-range accuracy requirement for track measurements; however, their research on track irregularities was generally based on analyzing the performance of the INS short-term accuracy and GNSS absolute accuracy. In the POS/TG system jointly developed by Applanix Inc. (Richmond Hill, Canada) and Plasser & Theurer Inc. (Wien, Austria) [7], a POS system together with other optical sensors, such as track gauge systems, were applied to obtain the position and attitude of track inspection trains and derive the geometric track parameters, but the related technology has not been made public. Zhu et al. [27] proposed an attitude variometric approach using double-differenced GNSS and INS integration to detect deformation in railway track irregularity measurements. A method of signal filter cut-off frequency determination was proposed to enhance the absolute accuracy of rail track irregularity detection and location [28]. The stand-alone GNSS/INS system is limited by the weak observability of the state variables under some conditions with insufficient dynamics, such as going straight for a long time [29]. The motion constraint, which does not require additional sensors, is a type of natural auxiliary information that can improve the integrated GNSS/INS navigation accuracy considering the dynamic characteristics of a carrier in practical applications [30].

The contribution of this paper is to provide an intuitive solution and theoretical support to answer why the centimeter-level positioning systems can achieve the millimeter-level precision measurement by the relationship the relative accuracy and track irregularity measurement. Since the relative measurement is the essence of the track irregularity measuring, we focus on the concept of the spatial relative accuracy of integrated GNSS/INS systems and deduce the relationship the relative accuracy and track irregularity measurement. This paper is structured as follows: Section 2 explains the concept of the GNSS/INS relative accuracy and different evaluation methods. Section 3 assesses the relative spatial accuracy of the studied GNSS/INS based on the allowable track irregularity. Section 4 illustrates the process of motion-constrained GNSS/INS integration. Section 5 and Section 6 give the experimental description and analysis of the field test results, respectively. Section 7 concludes the paper with a summary and an outlook regarding future work.

## 2. GNSS/INS Relative Spatial Accuracy

### 2.1. Concept of GNSS/INS Relative Spatial Accuracy

The term “accuracy” generally denotes a statistical measure that reflects the degree of conformance between estimated or measured parameters (e.g., position, velocity and/or attitude) at a given time (or position) and the reference parameters [11,31]. Accuracy is described best in terms of the absolute accuracy and relative accuracy to clarify the different representations. Absolute accuracy is generally expressed as the RMS error or standard deviation (STD) error and is the degree of departure from the reference values; it commonly represents the total navigation error, which is dominated by slowly varying error [11]. The current concern related to navigation accuracy mainly refers to the absolute accuracy, but this type of accuracy cannot reflect the level of relative variation among different points and times.

The relative accuracy described here considers the relevant relative variations of the navigation error at a given time or distance scale, and these variations can be between adjacent clusters or different points. Thus, the relative accuracy is a statistical measure of the relative variation, regardless of any error in the true navigation solution. The relative accuracy can reflect the stability, correlation and smoothness of the navigation error, and it is dominated by random error.

**Figure 1:**
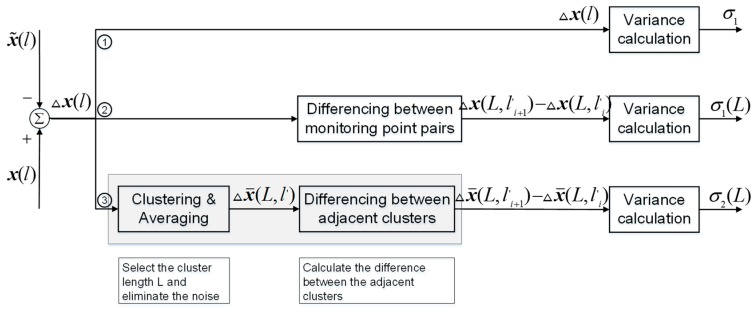
Comparison between different variance calculations based on three different variables.

Figure 1 gives three variables of interest for statistical analysis. The first variable is the navigation error Δx(l), which represents the difference between measured or estimated values $\tilde{x}\left(l\right)$ and the reference or true values x(l). Because Δx(l) includes all types of errors, including systematic error and random error, the statistical results of the total variation in the navigation error reflect the absolute accuracy. The corresponding statistical calculation of σ can be expressed as follows,

(1) $$\sigma =\sqrt{\frac{1}{N-1}{\sum_{i=1}^{N}\left[\Delta x\left(l\right)\right]}^{2}},$$

where l is the distance and N is the number of navigation error sequences.

The second variable reflects the relative variation $\Delta x\left(L,l_{i+1}^{\prime }\right)-\Delta x\left(L,l_{i}^{\prime }\right)$ of the navigation error Δx(l) between different points. $\Delta x\left(L,l_{i}^{\prime }\right)$ represents the navigation errors at the specified distance points, and L is the selected distance between different points. Because $\Delta x\left(L,l_{i+1}^{\prime }\right)-\Delta x\left(L,l_{i}^{\prime }\right)$ eliminates the systematic error and mainly contains the random error, the statistical results of the relative variation between different points reflect the relative spatial accuracy. The relative spatial accuracy considering the distance scale of different points is described in terms of $\sigma_{1}\left(L\right)$, which can be written as

(2) $$\sigma_{1}\left(L\right)=\sqrt{\frac{1}{N_{1}-1}{\sum_{i=1}^{N_{1}-1}\left[\Delta x\left(L,l_{i+1}^{\prime }\right)-\Delta x\left(L,l_{i}^{\prime }\right)\right]}^{2}},$$

where $N_{1}$ is the number of constructed sequences $\Delta x\left(L,l_{i}^{\prime }\right)$, $l_{}^{\prime }$ is the constructed distance based on L.

The third variable is the relative variation $\Delta \bar{x}\left(L,l_{i+1}^{\prime }\right)-\Delta \bar{x}\left(L,l_{i}^{\prime }\right)$ of the navigation error Δx(l) between adjacent clusters based on different distance scales L, where $\Delta \bar{x}\left(L,l_{}^{\prime }\right)$ represents the average navigation error of a specified cluster to eliminate the systematic error and weaken the effect of high-frequency noise. The spatial relative accuracy on the distance scales of different clusters is described in terms of $\sigma_{2}\left(L\right)$, which can be written as

(3) $$\sigma_{2}\left(L\right)=\sqrt{\frac{1}{N_{2}-1}{\sum_{i=1}^{N_{2}-1}\left[\Delta \bar{x}\left(L,l_{i+1}^{\prime }\right)-\Delta \bar{x}\left(L,l_{i}^{\prime }\right)\right]}^{2}},$$

where $N_{2}$ is the number of constructed sequences $\Delta \bar{x}\left(L,l_{i}^{\prime }\right)$.

As shown in Equations (1)–(3), the relationship between the three statistical standard deviations can be expressed as

(4) $$\sigma \geq \sigma_{1}\left(L\right)\geq \sigma_{2}\left(L\right).$$

It is important and convenient to clearly define the absolute accuracy and relative accuracy because in most cases, the navigation error is comprised of a slow-varying signal with almost no noise; additionally, in some applications, the accuracy of the change in navigation is most important (such as for track irregularities). As shown by the above description, the definition of the absolute accuracy is determinate, and the expression of relative accuracy differs. The relative accuracy depends on the specific application requirements. This paper will clarify the types of relative accuracy required for track irregularity measurement and attempt to establish the relationships among different relative accuracy expressions. In addition, it should be noted that the research on relative accuracy is carried out with continuous GNSS assistance to satisfy the high accuracy requirement of precision measurements. The scenarios of long term GNSS signal outages (e.g., long tunnel) are not considered because of the drifted position error without additional auxiliary information. An accuracy analysis is a statistical calculation process based on large sample data, and a small number of possible faults (e.g., multipath error) do not significantly affect the final statistical results based on the whole data.

### 2.2. Allan Variance

The Allan variance (AVAR), which is a method of evaluating the level of the relative variation between adjacent clusters, can be viewed as the application of a variable rectangular window to time series of data, and it was originally a time domain analysis technique used to study the frequency stability of precision oscillators and to characterize the error of inertial sensors [32,33]. Essentially, the AVAR method can be regarded as a stand-alone data analysis approach that can be applied in error analyses of instruments, and this approach is not limited to sensors but can be extended to entire systems. This method makes it possible to effectively study the characteristics of the noise component of data, such changes in the location of a station and the coordinates of radio sources [34,35]. Some research related to the GNSS/INS relative accuracy on different time scales has applied the AVAR method to evaluate the short-term accuracy [14].

For time series, the AVAR (or the corresponding square root, the Allan deviation; ADEV) represents the stability at different time scales for a set of sample data, and the time can be converted to distance using velocity information, so AVAR (or ADEV) at different distance scales can be obtained for a set of sample data. The expression of the Allan deviation $\sigma_{\mathrm{ADEV}}\left(L\right)$ is

(5) $$\sigma_{\mathrm{ADEV}}\left(L\right)=\sqrt{\frac{1}{2\left(N_{L}-1\right)}\sum_{k=1}^{N_{L}-1}{\left({\bar{x}}_{k+1}-{\bar{x}}_{k}\right)}^{2}},$$

where $\bar{x}$ is the average of sample data sequence x for a specific distance L and $N_{L}$ is the number of averages for each specific distance. The relationship between the Allan deviation $\sigma_{\mathrm{ADEV}}\left(L\right)$ and the third standard deviation $\sigma_{2}\left(L\right)$ in Equation (3) is as follows.

(6) $$\sigma_{\mathrm{ADEV}}\left(L\right)=\frac{\sigma_{2}\left(L\right)}{\sqrt{2}}$$

This paper determines the relationships between the different standard deviations of the integrated navigation errors and the measurement tolerance of track irregularities to verify that the relative spatial accuracy of integrated navigation meets the requirements of track irregularity assessment.

## 3. Relationship between Relative Spatial Accuracy and Track Irregularities

### 3.1. Evaluation Indicator of Track Irregularities

**Figure 2:**
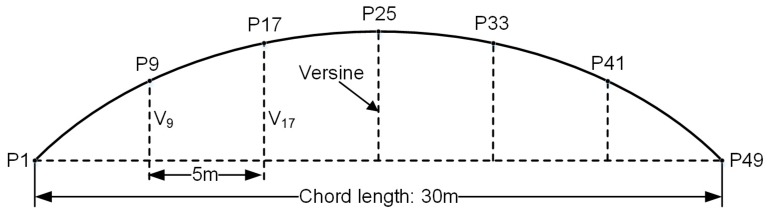
Principle of the shortwave track irregularity measurement with a chord length of 30 m.

The relative accuracy can be verified by measurements of versine along a chord, and track irregularity measurements are based on a concept that the track curvature can be determined by the versine of the chord [1]. A chord length of 20–30 m is usually applied in shortwave track irregularity measurements, and a long wave based on a chord length of 300 m is commonly used to detect long periodic patterns, such as subgrade settlement and bending deflection [1,36]. Here, a measurement configuration with 30 m chord length and 5 m spacing division is used as an example to illustrate the process of shortwave track irregularity measurement, as shown in Figure 2 [12,36]. It should be noted that the definition of the shortwave in this paper is mainly for the track irregularity according to the inspecting instrument for China railway track, not the wear of the rail.

Figure 2 shows a shortwave track irregularity measured with a chord length of 30 m and a spacing division of 5 m [36]. The points from P1 to P49 denote the serial numbers of the railway sleepers at the central line. V9 and V17 are the versines, representing the distances from points P9 and P17 to the chord P1–P49, respectively. Assuming that the distance between railway sleepers is 0.625 m, the distance between a pair of monitoring points is chosen as 5 m, which is exactly eight times longer than the distance between railway sleepers. The evaluation indicator ΔV of shortwave track irregularity measured with a chord of 30 m in length can be expressed as

(7) $$\Delta V=\left|\left(V_{design,9}-V_{design,17}\right)-(V_{measured,9}-V_{measured,17})\right|,$$

where $V_{design,9}$ and $V_{design,17}$ are the designed distances from points P9 and P17 to the chord line P1P49, respectively, and they are also called the designed versines. $V_{real,9}$ and $V_{real,17}$ are the corresponding measured versines. ΔV represents the difference between the designed relative versines and the measured relative versines for pairs of monitoring points, and this value is usually converted to the distance between monitoring points.

Considering the versine difference of each point, ΔV can be rewritten as follows:

(8) $$\Delta V=\left|\left(V_{design,9}-V_{measured,9}\right)-(V_{design,17}-V_{measured,17})\right|=\left|\Delta V_{measured,9}-\Delta V_{measured,17}\right|,$$

where $\Delta V_{measured,9}$ and $\;\Delta V_{measured,17}$ are the versine differences for points P9 and P17, respectively.

In practice, a measured versine is obtained by a projection from horizontal coordinates considering the elevation to the corresponding chord line. Equation (8) indicates that the evaluation indicator highly depends on the relative variation in the positioning error. Therefore, the relative position relationships of monitoring points considerably affect track deformation. The evaluation indicator ΔV does not equal zero if the measured versines are not consistent with the designed values, which reflects track deformation. Table 1 lists the allowable values of the evaluation indicators for shortwave and longwave track irregularity measurement, which is applied in the China railway regulation [36].

### 3.2. Assessment of Relative Spatial Accuracy in Track Irregularity Measurement

**Figure 3:**
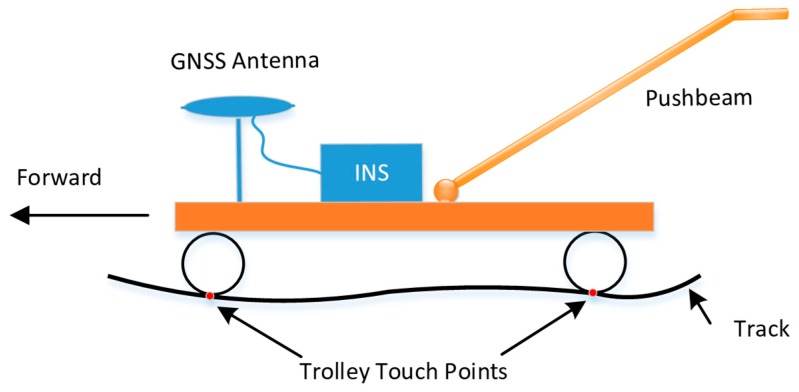
Track geometry measurement trolley based on a Global Navigation Satellite System/Inertial Navigation System (GNSS/INS) system.

An integrated GNSS/INS has the relative measurement ability of INS, and it can sense small changes in navigation information, such as position and attitude variations between adjacent points. In theory, an integrated GNSS/INS has the relative measurement ability required for track irregularity measurement. Figure 3 shows the trolley used for track geometry measurements based on a GNSS/INS system [4]. The GNSS/INS system is rigidly mounted on the trolley frame, and the trolley wheels maintain a rigid connection with the rails. Track irregularity will cause changes in trolley motion in kinematic surveying mode, and these changes can be sensed and measured by the GNSS/INS system fixed on the trolley. Moreover, track irregularities are determined by the relative position relationships among monitoring points. Therefore, the ability of the GNSS/INS to measure track irregularities can be converted to a spatial relative accuracy in coordinate form.

**Figure 4:**
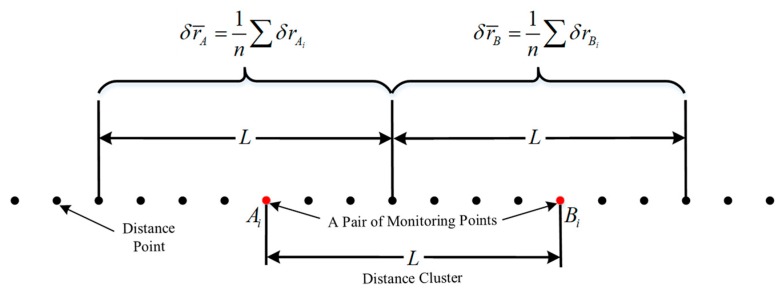
Illustration of the relative position relationships for different distance points.

The spatial relative accuracy of a GNSS/INS at different distance scales is analyzed through the relative position relationships of various points. Figure 4 illustrates the relative position relationships for two distance points. $A_{i}$ and $B_{i}$ are the arbitrary track distance points in the adjacent distance clusters with a given distance length L, and L (i.e., 5 m) represents the distance length between $A_{i}$ and $B_{i}$, which is the distance between monitoring points used in the track irregularity measurements. $\delta r_{A_{i}}$ and $\delta r_{B_{i}}$ are the corresponding position errors at the points $A_{i}$ and $B_{i}$, which influence the measured versine obtained by the projection from the coordinates to the specified chord line. $\delta {\bar{r}}_{A}=\frac{1}{n}\sum_{i=1}^{n}\delta r_{A_{i}}$ and $\delta {\bar{r}}_{B}=\frac{1}{n}\sum_{i=1}^{n}\delta r_{B_{i}}$ represent the means of the position errors at the distance points in the specified distance clusters and they are constructed for building the relationship between the Allan variance and the allowable deviation in the shortwave track irregularity measurements, and n is the number of distance points.

The versine is calculated from the coordinates of distance points and the measurement error of versine is equivalent to the positioning error of the distance points provided by position sensors [1,4]. Therefore, as shown in Equation (8), the relative variation in the position errors of the monitoring points should be less than the allowable deviation in the shortwave track irregularity measurements, so the following relationship can be obtained.

(9) $$\left|\delta r_{B_{i}}-\delta r_{A_{i}}\right|\;\leq \;2\;mm$$

To meet the requirement of the allowable deviation in shortwave track irregularity in the absolute track surveying, the root mean square error (MSE) of the relative variation should be one-third of the allowable deviation according to three-sigma rule of thumb [37], that is,

(10) $$\sigma_{\left(\delta r_{B_{i}}-\delta r_{A_{i}}\right)}=\sqrt{\frac{1}{N-1}\sum_{i=1}^{N-1}{\left(\delta r_{B_{i}}-\delta r_{A_{i}}\right)}^{2}}\;\leq \;\frac{1}{3}\;\times \;2\;mm\approx 0.67\;mm,$$

where N represents the total number of distance points.

Equation (10) shows that the MSE can directly provide the degree of relative variation in the position errors between two points, so it can be used to evaluate the relative measurement ability of a GNSS/INS in the track irregularity measurement. The MSE at a 5 m distance should be no more than 0.67 mm to meet the accuracy requirement of shortwave irregularity measurements.

In addition, we can derive the tolerance threshold of the AVAR of the position errors through the MSE to establish the relationship between the relative spatial accuracy and the allowable deviation in track irregularity. Here, the variable $\Delta \delta \bar{r}$, which represents the difference in the average position errors between adjacent distance clusters, as shown in Figure 4, is defined as follows:

(11) $$\Delta \delta \bar{r}\;=\;\frac{1}{n}\sum_{i=1}^{n}\delta r_{B_{i}}-\;\frac{1}{n}\sum_{i=1}^{n}\delta r_{A_{i}}=\;\frac{1}{n}\sum_{i=1}^{n}\left(\delta r_{B_{i}}-\;\delta r_{A_{i}}\right),$$

where n represents the number of distance points in a specified range.

In mathematics, the inequality of arithmetic and quadratic means states that the quadratic mean of a list of real numbers is greater than or equal to the arithmetic mean of the same list, that is, for any list of m numbers $x_{1},x_{2},\cdots x_{i},\cdots ,x_{m}$ ($x_{i}\in \mathbb{R},\;i\geq 1,\;m\geq 1$), the relationship between the arithmetic mean and quadratic mean can be represented as follows.

(12) $$\frac{x_{1}+x_{2}+\cdots +x_{m}}{m}\leq \sqrt{\frac{x_{1}{}^{2}+x_{2}{}^{2}+\cdots +x_{m}{}^{2}}{m}}\;\Rightarrow \;{\left(\frac{1}{m}\sum_{i=1}^{m}x_{i}\right)}^{2}\leq \frac{1}{m}\sum_{i=1}^{m}x_{i}{}^{2}$$

According to Equation (12), $\Delta \delta \bar{r}$ meets the following condition:

(13) $$\Delta \delta {\bar{r}}^{2}={\left(\frac{1}{n}\sum_{i=1}^{n}\left(\delta r_{B_{i}}-\delta r_{A_{i}}\right)\right)}^{2}\leq \frac{1}{n}\sum_{i=1}^{n}{\left(\delta r_{B_{i}}-\delta r_{A_{i}}\right)}^{2}.$$

The AVAR expression of the GNSS/INS navigation error in track irregularity measurement can be expressed by the following formula.

(14) $$\sigma_{ADEV}{\left(L\right)}^{2}=\frac{1}{2\left(N_{L}-1\right)}\sum_{k=1}^{N_{L}-1}{\left({\left(\frac{1}{n}\sum_{i=1}^{n}\delta r_{B_{i}}\right)}_{k+1}-{\left(\frac{1}{n}\sum_{i=1}^{n}\delta r_{A_{i}}\right)}_{k}\right)}^{2}$$

Here, the position error is assumed to be a steady-state random process to ensure that the deviation does not diverge with increasing sample size. By combining Equations (13) and (14), one-third of $\sigma_{\left(\delta r_{B_{i}}-\delta r_{A_{i}}\right)}^{}$ is selected to meet the sufficient conditions for track irregularity measurement. Therefore, we can obtain the allowable AVAR of the navigation errors for track irregularity measurements as follows.

(15) $$\sigma_{ADEV}{\left(L\right)}^{2}\leq \frac{1}{2}\;\times \;{\left(\frac{1}{3}\;\times \;\sigma_{\left(\delta r_{B_{i}}-\delta r_{A_{i}}\right)}^{}\right)}^{2}$$

Here, a shortwave track irregularity measurement is taken as an example, and the allowable Allan deviation should be as follows.

(16) $$\sigma_{ADEV}\left(L=5\right)\leq \frac{\;1}{\sqrt{2}}\;\times \;\frac{1}{3}\;\times \;0.67\;mm\approx 0.16\;mm$$

In summary, the level of relative spatial accuracy is the prerequisite for applications involving track irregularity detection, and the MSE and Allan deviation at a 5-m distance scale need be less than or equal to 0.67 mm and 0.16 mm, respectively, to meet the requirements of shortwave track irregularity detection. However, the derivation in the threshold value of the Allan deviation is based on the steady-state assumptions and high reliability conditions, so this value may be difficult to determine. In the following section, we will analyze and verify the relative spatial accuracy of integrated GNSS/INS navigation at different distance scales through field tests.

## 4. Motion-Constrained GNSS/INS integration

For GNSS/INS integration, an integration algorithm with 21 states is used. The error state vector can be expressed as $\delta x={\left[\begin{array}{ccccccc} \delta r^{T} & \delta v^{T} & \phi^{T} & b_{\omega }{}^{T} & b_{f}{}^{T} & s_{\omega }{}^{T} & s_{f}{}^{T} \end{array}\right]}^{T}$, which is given by the navigation error states, including the position error $\delta r={\left[\begin{array}{ccc} \delta r_{N} & \delta r_{E} & \delta r_{D} \end{array}\right]}^{T}$, velocity error $\delta v={\left[\begin{array}{ccc} \delta v_{N} & \delta v_{E} & \delta v_{D} \end{array}\right]}^{T}$, attitude error $\phi ={\left[\begin{array}{ccc} \phi_{roll} & \phi_{pitch} & \phi_{heading} \end{array}\right]}^{T}$, and IMU error, which encompasses the biases ($b_{\omega }$ and $b_{f}$) and scale factors ($s_{\omega }$ and $s_{f}$) of the gyroscopes and accelerometers. A complete system model of GNSS/INS integration, including the dynamic INS error equation based on ϕ -angle error model (it is built with respect to the true navigation frame) and the sensor error model based on the first Gauss-Markov process, can be written as follows [38,39]:

(17) $$\begin{array}{l} \delta {\dot{r}}^{n}=-\omega_{en}^{n}\times \delta r^{n}+\delta \theta \times v^{n}+\delta v^{n}\\ \delta {\dot{v}}^{n}=C_{b}^{n}\delta f^{b}+C_{b}^{n}f^{b}\times \phi -\left(2\omega_{ie}^{n}+\omega_{en}^{n}\right)\times \delta v^{n}+v^{n}\times \left(2\delta \omega_{ie}^{n}+\delta \omega_{en}^{n}\right)+\delta g^{n}\\ \dot{\phi }\;=\;-\omega_{in}^{n}\times \phi -C_{b}^{n}\delta \omega_{ib}^{b}+\delta \omega_{in}^{n}\\ {\dot{b}}_{\omega }\;=\;-\frac{1}{T_{b\omega }}b_{\omega }+w_{b\omega }\;\\ {\dot{b}}_{f}\;=\;-\frac{1}{T_{bf}}b_{f}+w_{bf}\;\\ {\dot{s}}_{\omega }\;=\;-\frac{1}{T_{s\omega }}s_{\omega }+w_{s\omega }\;\\ {\dot{s}}_{f}\;=\;-\frac{1}{T_{sf}}s_{f}+w_{sf}\; \end{array}$$

where all parameters are with respect to the navigation frame. All symbols are defined as follows: $\delta \theta \;={\left[\begin{array}{ccc} \delta r_{E}/\left(R_{n}+h\right) & -\delta r_{N}/\left(R_{m}+h\right) & -\delta r_{E}\tan\varphi /\left(R_{n}+h\right) \end{array}\right]}^{T}$, which is a rotation vector describing the misalignment of the computed frame with respect to the true navigation frame; $R_{m}$ and $R_{n}$ are the radiuses of curvature in the meridian and the prime vertical, respectively; h is the height; φ is the local geodetic latitude; $\delta {\dot{r}}^{n}$, $\delta {\dot{v}}^{n}$ and $\dot{\phi }$ are the time derivatives of position error, velocity error and attitude error; $C_{b}^{n}$ represents the rotation matrix from the body frame (*b*) to the navigation frame (*n*); $\omega_{en}^{n}$, $\omega_{ie}^{n}$ and $\omega_{in}^{n}$ represent the angle rate of the navigation frame relative to the Earth frame (*e*), the Earth frame relative to the inertial frame (*i*), and the navigation frame relative to the inertial frame, respectively, and $\delta \omega_{en}^{n}$, $\delta \omega_{ie}^{n}$ and $\delta \omega_{in}^{n}$ are the corresponding angular rate errors; $f^{b}$ is the specific force on the body frame; $\delta g^{n}$ is the normal local gravity error; and $\delta f^{b}$ and $\delta \omega_{ib}^{b}$ represent the sensor errors of the accelerometers and gyroscopes; ${\dot{b}}_{\omega }$ and ${\dot{b}}_{f}$ are the time derivations of the bias of gyro and accelerometer; ${\dot{s}}_{\omega }$ and ${\dot{s}}_{f}$ are the time derivations of the bias of gyro and accelerometer; $T_{b\omega }$, $T_{bf}$, $T_{s\omega }$ and $T_{sf}$ represent the correlation time of the bias and scale factor of gyro and accelerometer, respectively; $w_{b\omega }$, $w_{bf}$, $w_{s\omega }$ and $w_{sf}$ represent the driven white noise of the bias and scale factor of gyro and accelerometer, respectively.

The position and velocity of GNSS antenna is related to the INS solution by taking into account the lever arm as follows [40]:

(18) $$\begin{array}{l} r_{GNSS}^{n}=r_{IMU}^{n}+D_{R}{}^{-1}C_{b}^{n}l_{GNSS}^{b},\;D_{R}{}^{-1}=\left[\begin{array}{ccc} 1/\left(R_{m}+h\right) & & \\ & 1/\left(R_{n}+h\right)\cos\varphi & \\ & & 1 \end{array}\right]\\ v_{GNSS}^{n}=v_{IMU}^{n}-\left(\omega_{in}^{n}\times \right)C_{b}^{n}l_{GNSS}^{b}-C_{b}^{n}\left(l_{GNSS}^{b}\times \omega_{ib}^{b}\right) \end{array}$$

where $r_{GNSS}^{n}$ and $r_{IMU}^{n}$ are the positions of the GNSS antenna phase center and the IMU measurement center; $l_{GNSS}^{b}$ is the lever arm from the IMU measurement center to the GNSS antenna phase center resolved in the body frame; $D_{R}{}^{-1}$ refers to the Cartesian-to-curvilinear position change transformation matrix; $v_{GNSS}^{n}$ and $v_{IMU}^{n}$ are the velocities of the GNSS antenna phase center and the IMU measurement center.

Hence, the measurement models based on GNSS position and velocity can be expressed as [40]

(19) $$\begin{array}{l} z_{r_{GNSS}}=\delta r_{}^{n}+\left(C_{b}^{n}l_{GNSS}^{b}\times \right)\phi -e_{r}\\ z_{v_{GNSS}}=\delta v_{}^{n}-\left(\omega_{in}^{n}\times \right)C_{b}^{n}\left(l_{GNSS}^{b}\times \right)\phi -C_{b}^{n}\left(l_{GNSS}^{b}\times \omega_{ib}^{b}\right)\times \phi -C_{b}^{n}\left(l_{GNSS}^{b}\times \right)\delta \omega_{ib}^{b}-e_{v} \end{array}$$

where $z_{r_{GNSS}}$ and $z_{v_{GNSS}}$ are the constructed position error vector and velocity error, respectively; $e_{r}$ and $e_{v}$ are the observation noise vector of the GNSS positon and velocity, respectively.

The relationship between the trolley wheel velocity and the IMU velocity can be built through the lever arm that represents the spatial position relation between the trolley wheel and the IMU center. Hence, the trolley wheel velocity can be expressed as

(20) $$v_{wheel}^{v}=C_{b}^{v}C_{n}^{b}v_{imu}^{n}+C_{b}^{v}\left(\omega_{nb}^{b}\times \right)l_{wheel}^{b},$$

where $C_{b}^{v}$ is the rotation matrix from the body frame to the vehicle frame (*v*); $C_{n}^{b}$ represents the rotation matrix from navigation frame the to the body frame; $l_{wheel}^{b}$ is the lever arm from the IMU measurement center to the point at which the trolley wheels touch the rails, which is resolved in the body frame; $\omega_{nb}^{b}$ represent the angle rate of the body frame relative to the navigation frame in the body frame. The estimated velocity at the wheel point is denoted as ${\hat{v}}_{wheel}^{v}={\left[\begin{array}{ccc} {\hat{v}}_{wheel,x}^{v} & {\hat{v}}_{wheel,y}^{v} & {\hat{v}}_{wheel,z}^{v} \end{array}\right]}^{T}$.

Since the dynamic characteristics of the track geometry measurement trolley are insufficient, the estimation of the system state (especially the heading estimation) may be poor when only auxiliary measurements from the GNSS are available. Therefore, motion constraints can be applied to enhance GNSS/INS integration. In railway track surveying applications, the wheels of the track trolley are designed to maintain reliable and continuous rigid contact with the rails when moving. Thus, the motion of the track trolley on the rails is governed by two non-holonomic constraints (NHCs) because the trolley does not jump off the rails or slide on the rails. In this case, the velocities of the trolley in both cross-track directions are zero. Hence, the lateral and vertical velocity measurements in the vehicle frame can be expressed as follows [30]:

(21) $$\begin{array}{l} v_{y}^{v}\approx 0\\ v_{z}^{v}\approx 0 \end{array}$$

where $v_{y}^{v}$ and $v_{z}^{v}$ represent the velocity of the vehicle in the plane perpendicular to the forward direction (x-axis). The NHCs can be used for velocity measurement updating with a Kalman filter to enhance the navigation accuracy and reliability.

The NHC velocity error measurement equation in the vehicle frame can be expressed as

(22) $$z_{v_{wheel}}=\left[\begin{array}{c} {\hat{v}}_{wheel,y}^{v}-v_{y}^{v}\\ {\hat{v}}_{wheel,z}^{v}-v_{z}^{v} \end{array}\right]={\left(C_{b}^{v}C_{n}^{b}\delta v^{n}-C_{b}^{v}C_{n}^{b}\left(v^{n}\times \right)\phi -C_{b}^{v}\left(l_{wheel}^{b}\times \right)\delta \omega_{ib}^{b}\right)}_{y,z}+\left[\begin{array}{c} \eta_{y}\\ \eta_{z} \end{array}\right],$$

where $z_{v_{wheel}}$ represents the velocity error between the estimated velocity $\left({\hat{v}}_{wheel,y}^{v},{\hat{v}}_{wheel,z}^{v}\right)$ and the constrained velocity $\left(v_{y}^{v},v_{z}^{v}\right)$ in the vehicle frame; $\eta_{y}$ and $\eta_{z}$ represent the noise associated with lateral and vertical velocity measurements, respectively; and the symbol ${\left(\right)}_{y,z}$ represents a two-dimensional vector that consists of the second and third rows of the three-dimensional vector.

**Figure 5:**
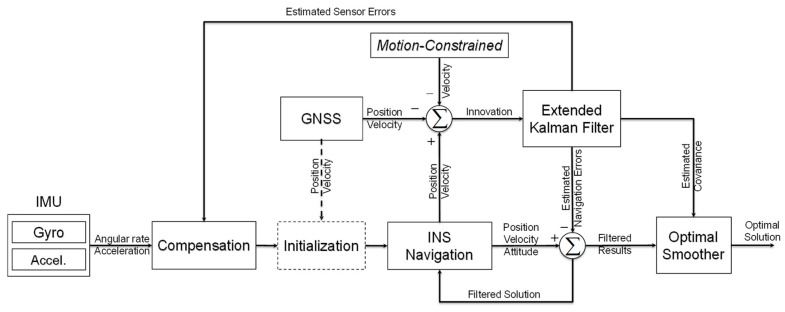
Flowchart of motion-constrained GNSS/INS integration for the track irregularity measurement.

Figure 5 gives the flowchart of motion-constrained GNSS/INS integration. A brief description of data fusion is summarized as follows [4,39].

▪ Error compensation: The outputs of the inertial sensors (i.e., gyroscopes and accelerometers) should first be corrected with the sensor errors before they are input into the navigation algorithm. The raw IMU measurements can be adjusted online by the estimated sensor errors from the optimal estimation.
▪ Navigation initialization: This process, marked by the dotted line in Figure 5, mainly provides the initial attitude from different alignment methods to ensure satisfactory initial navigation accuracy. The process is generally executed once if there is no navigation restart. The initial position and velocity can be obtained by the GNSS or be given manually.
▪ INS navigation: The compensated acceleration measurements are rotated and integrated to update the INS velocity and position, and the INS attitude is calculated from the compensated gyroscope measurements. This process is usually called INS mechanization.
▪ Motion constraint: The constrained motion of the track trolley on the rails discussed in this section is taken as additional virtual velocity information to enhance the integrated navigation estimation.
▪ Kalman filter: The navigation system will pass a received position and/or velocity information obtained from auxiliary sensors or some constraints to the extended Kalman filter to update measurements.
▪ Optimal smoother: Considering the high precision requirements in the post processing applications, an optimal smoother (e.g., RTS smoother) is applied to restrain the INS drift error between each correction of the auxiliary information (e.g., GNSS) and achieve the highest possible accuracy and smoothest navigation results.

## 5. Experimental Description

### 5.1. Description of the Situation and Equipment

**Figure 6:**
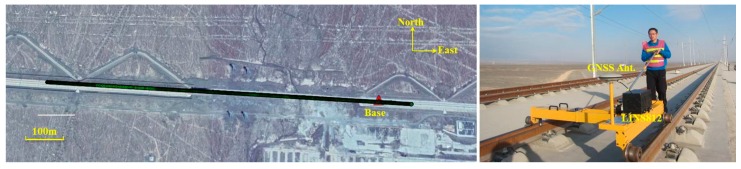
Field test trajectory (**left**) and track geometry measurement trolley (**right**).

To evaluate the absolute accuracy and relative spatial accuracy of integrated GNSS/INS navigation for a track measurement system, a field test was conducted in November 2013 in the Turpan-Shanshan section of the second Lanzhou-Xinjiang high-speed railway, as shown in Figure 6. The test track, roughly situated in the east-west direction, is a ballastless track under construction and is a straight-line segment of approximately 1.0 km in length. The test area is in an open-sky environment, and the GNSS signal is unobstructed to provide relatively good GNSS observation conditions. A GNSS base station (Trimble NetR9 receiver (Sunnyvale, CA, USA)) was set up near the test track for postprocessing in carrier-phase differential GNSS mode, which has a short baseline and ensures an accurate GNSS solution. During the data collection stage, the section of track was repeatedly measured three times (in turn, named ch1, ch2 and ch3), and there was no trolley moved; specifically, the push rod was only pushed in opposing directions in round trip cycles. The trolley was pushed at a speed of approximately 3 m/s by human force.

The track geometry measurement trolley developed by Wuhan University, as shown in Figure 6, was used for GNSS and IMU data acquisition. A navigation-grade GNSS/INS system called LINS812 was tightly mounted to the trolley. The specifications of this system are listed in Table 2.

### 5.2. Description of the Reference Information

**Figure 7:**
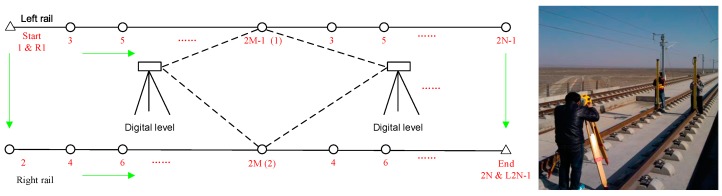
Schematic of the levelling survey based on the Trimble DiNi (**left**) and test scenes (**right**).

A high-precision digital level and an automatic measurement total station were used in this experiment to provide the reference information in the vertical and horizontal directions, respectively, and obtain the spatial error sequence of integrated GNSS/INS navigation. A Trimble DiNi digital level was used to measure the heights of both rails at each sleeper point with a 0.625-m distance interval. The relative accuracy measured by the digital level is approximately 0.3 mm after error adjustment, which is accurate enough to establish a reference and evaluate the surveying accuracy of the GNSS/INS in the vertical direction. Figure 7 shows a schematic of the levelling survey. It should be noted that the height measurements only cover a distance of approximately 600 m because the workload of the levelling survey is relatively large, and the process is complex.

An Amberg Slab Track GRP1000 system (Regensdorf-Wattcity, Switzerland) is capable of automatically identifying prisms mounted on the trolley. The coordinates of unknown points can be defined by ranging the intersections and establishing the free stations of the total station. Additionally, some of the control points located at adjacent stations were used to unify the coordinate systems. The GRP1000 system can provide an absolute track position accuracy of up to 1.0 mm in stop-and-go mode for multiple measurements, and it can provide a relative track geometry (versine) accuracy of ±0.7 mm (2-sigma) for shortwave track irregularity measuring. Although taking GRP1000 system as a reference system may affect the reliability of horizontal position accuracy evaluation, the smaller the position difference between the tested system and the GRP1000 system is, the better the performance of the tested system.

## 6. Results and Discussion

Because the relative spatial accuracy of GNSS/INS integration based on different distance scales is the focus of this integrated navigation accuracy assessment, the position error on the horizontal axis is given as a distance. Here, the results related to the accuracy requirements of shortwave track irregularities are presented considering the limited length of the tested track. Additionally, it should be noted that the vertical and lateral position errors are analyzed because the test track is mainly oriented in the east-west direction and the east position error minimally affects the vertical and alignment irregularities of the track.

### 6.1. Results of GNSS/INS Mode

**Figure 8:**
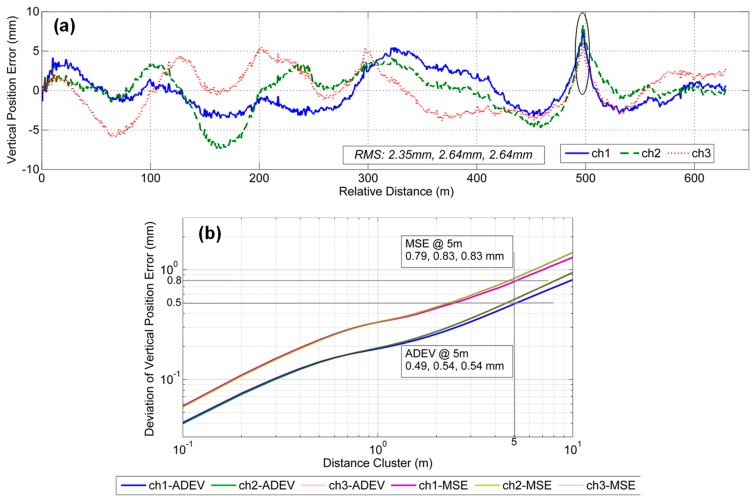
Vertical position error results in GNSS/INS integration mode with two evaluation deviations: (**a**) vertical position errors and (**b**) the corresponding deviations including mean square error (MSE) and Allan deviation (ADEV).

Figure 8a shows the vertical position errors of GNSS/INS integration. The horizontal axis represents the relative distance after the initial distance is deducted. There are three vertical error curves corresponding to three repeated measurements for ch1, ch2 and ch3. It is clear that the vertical position errors change slowly with values mainly within the range of ±10 mm, and there is no obvious high-frequency noise. The absolute RMS of the vertical position error of each survey is approximately 2.35 mm, 2.64 mm and 2.64 mm. However, there is an incorrect jump marked in Figure 8a, which is mainly caused by uneven welding at the rail interface. However, the absolute vertical accuracy of GNSS/INS integration is does not meet the accuracy requirement of the shortwave vertical irregularities of the track because the relative accuracy is the focus of track irregularity measurements and the absolute statistical values do not reflect the relative relationships between different distance points.

Figure 8b shows the plots of the MSE and Allan deviation of the vertical position errors of GNSS/INS integration. The horizontal axis represents different distance clusters, and the largest distance scale is set as 10 m to ensure high-accuracy standard deviation results due to the limited length of the test track. The shorter the distance cluster is, the smaller the MSE and the Allan deviation, and the better the relative accuracy. Here, shortwave track irregularities are a concern, so we mainly focus on deviations based on the distance scale of 5 m. The corresponding MSE values are 0.79 mm, 0.83 mm, and 0.83 mm, which are larger than the threshold value of 0.67 mm, as shown in Equation (10). The ADEV values are 0.49 mm, 0.54 mm, and 0.54 mm, which are larger than the threshold value of 0.16 mm, as shown in Equation (16). The deviation difference of the three measurements can be controlled at less than 10%. The results indicate that the relative measurement capability of the GNSS/INS cannot fully meet the relative accuracy requirements of shortwave vertical irregularities.

**Figure 9:**
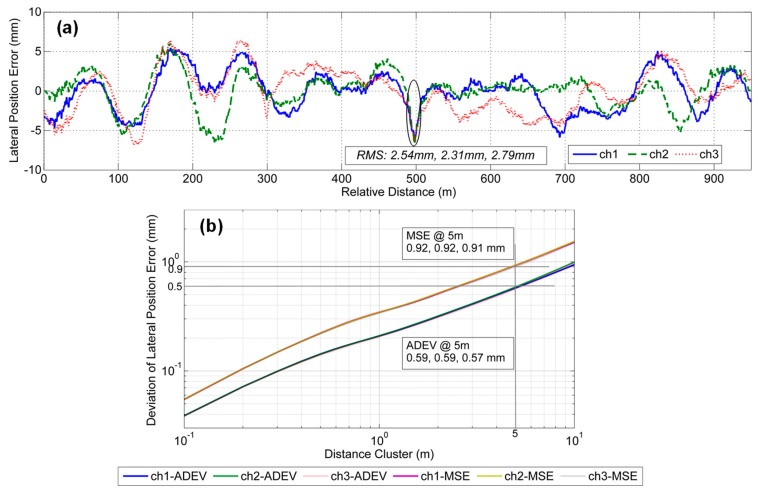
Lateral position error results in GNSS/INS integration mode with two evaluation deviations: (**a**) lateral position errors and (**b**) the corresponding deviations including MSE and ADEV.

Figure 9a shows the lateral position errors of GNSS/INS integration. It is clear that the lateral position errors are largely within ±10 mm; the absolute accuracy of each survey based on the RMS values is approximately 2.54 mm, 2.31 mm, and 2.79 mm. The error curves of the lateral position are not as stable as those of the vertical, as shown in Figure 8a, because the weak observability of the heading has a notable influence on the lateral position accuracy. This finding indicates that the relative accuracy in the lateral direction might be worse than that for the vertical. There is also an incorrect jump in the lateral position errors marked in Figure 9a because of the uneven welding at the rail interface. Compared to the absolute accuracy of the vertical, the accuracy of the absolute lateral position based on the GNSS/INS does not satisfy the accuracy requirements of the shortwave alignment irregularities of the track.

Figure 9b shows the plots of the MSE and Allan deviation for the lateral position errors of GNSS/INS integration. The MSE values are 0.92 mm, 0.92 mm, and 0.91 mm, and the ADEV values are 0.59 mm, 0.59 mm, and 0.57 mm. The deviation difference of the three measurements can be controlled at less than 5%. The deviations in the lateral position error are larger than those for the vertical and are generally consistent with the error curves. The results indicate that the relative measurement capability of GNSS/INS integration cannot meet the relative accuracy requirements of shortwave alignment irregularities.

As demonstrated by the above results, GNSS/INS integration cannot satisfy the accuracy requirements of shortwave track irregularities because there is not enough dynamic data to provide strong observations for optimal estimations. In this case, the motion constraints, such as the NHCs, were utilized to increase the integrated accuracy considering the motion characteristics of the trolley. The results of motion-constrained GNSS/INS integration are given below.

### 6.2. Results for Motion-Constrained GNSS/INS Mode

**Figure 10:**
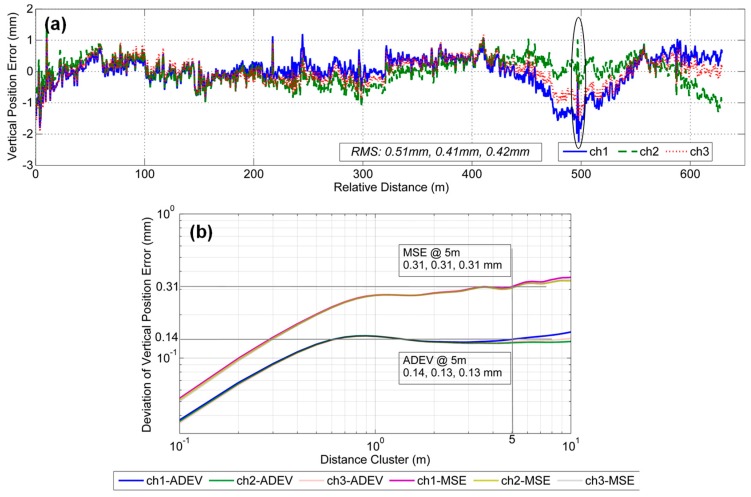
Vertical position error results in motion-constrained GNSS/INS integration mode with two evaluation deviations: (**a**) vertical position errors and (**b**) the corresponding deviations including MSE and ADEV.

Figure 10a shows the vertical position errors of motion-constrained GNSS/INS integration. The errors mainly remain within the range of ±2 mm; the absolute vertical accuracy of each survey based on the RMS values is approximately 0.51 mm, 0.41 mm and 0.42 mm, which are better than the values in Figure 8a. Figure 10b shows the plots of the MSE and Allan deviation of the vertical position errors of motion-constrained GNSS/INS integration. The MSE values are all 0.31 mm, and the ADEV values are 0.14 mm, 0.13 mm and 0.13 mm. The deviation difference of three measurements can be controlled at less than 5%. These values are smaller than the threshold values of 0.67 mm and 0.16 mm. Compared to the results in Figure 8b, there is a significant reduction in the deviations. The results indicate that the relative measurement capability of motion-constrained GNSS/INS integration meets the relative accuracy requirements of shortwave vertical irregularities.

**Figure 11:**
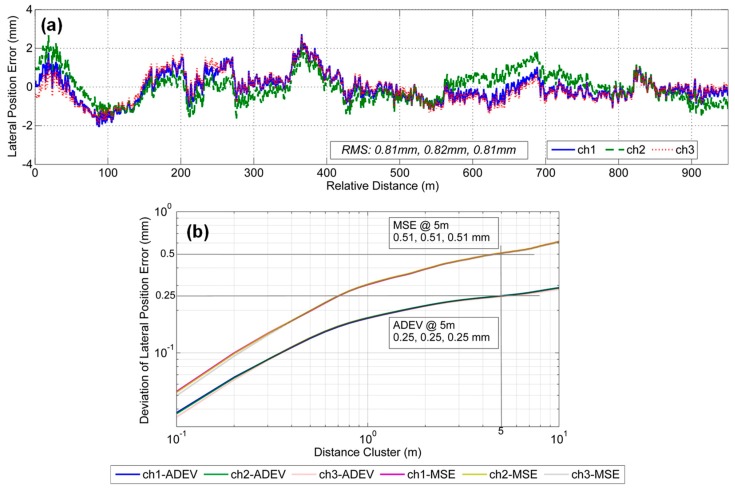
Lateral position error results in motion-constrained GNSS/INS integration mode with two evaluation deviations: (**a**) lateral position errors and (**b**) the corresponding deviations including MSE and ADEV.

Figure 11a shows the lateral position errors of motion-constrained GNSS/INS integration. The lateral position errors mainly remain within the range of ±2 mm, and the absolute lateral position accuracy of each survey based on the RMS values is approximately 0.81 mm, 0.81 mm and 0.82 mm. It can be seen that the lateral position errors of motion-constrained GNSS/INS integration is basically at the same level as the track position accuracy provided by GRP1000 system (approximately 1 mm), and it indicates that the accuracy level of the lateral position in this mode may not be reliably determined. But it can show that the lateral position error decreased significantly by the motion constraints compared with the results shown in Figure 9a. Figure 11b shows the deviation plots of the lateral position errors. It is clear that the MSE values are all 0.51 mm and are smaller than the threshold value of 0.67 mm; additionally, the ADEV values are all 0.25 mm and are a little larger than the threshold value of 0.16 mm. The results show that there are some slowly varying error affecting the relative accuracy, which may be caused by the stop-and-go mode of the total station or the weak observability of motion-constrained GNSS/INS integration resulting from strong coupling with the heading. However, the relative measurement capability of the motion-constrained GNSS/INS meets the relative accuracy requirements of shortwave alignment irregularities according to the MSE threshold value.

In conclusion, the stand-alone GNSS/INS cannot meet the accuracy requirements of the shortwave vertical and alignment irregularities of the track because of the weak observability of information resulting from limited dynamics. The motion constraint can enhance GNSS/INS integration and increase the position accuracy, thereby meeting the accuracy requirements of shortwave track irregularities, especially vertical irregularities. Table 3 lists the relative accuracy levels of the shortwave track irregularity measurements.

## 7. Conclusions

The concept of the relative spatial accuracy of a GNSS/INS is investigated in this paper by comparing the difference from the absolute accuracy. Two methods, including the standard deviation based on different points and Allan deviation based on different clusters to evaluate the relative spatial accuracy, were given. A requirement assessment of the relative spatial accuracy of the GNSS/INS in track irregularity measurement was performed based on an evaluation indicator of track irregularity, and the threshold values of 0.67 mm for the MSE and 0.16 mm for the ADEV of the relative spatial accuracy, which satisfy the constraints for shortwave track irregularities of China railway regulation, were derived. Motion-constrained GNSS/INS integration was performed to provide accuracy enhancement considering the dynamic characteristics of the track geometry measurement trolley.

The results of the field test show that the MSE and ADEV of the vertical and lateral position errors of GNSS/INS integration are larger than the threshold values because of the weak observability of state variables. Motion-constrained GNSS/INS integration can improve the relative accuracy and meet the accuracy requirements of shortwave track vertical irregularities. These conclusions can provide guidance for the application of the GNSS/INS system in track irregularity assessments of Chinese high-speed railway. Next, we will focus on introducing some low-cost sensors, such as odometers and laser scanners, to meet the application requirements of scenarios involving poor or no GNSS signals.

## Figures and Tables

**Table 1:** Allowable deviation in track irregularity measurement of China railway regulation.

Parameter	Wave	Chord Length (m)	Distance of the Monitoring Points (m)	Allowable Deviation (mm)
Track irregularity	Shortwave	30	5	2
	Longwave	300	150	10

**Table 2:** Specifications of the tested LINS812 system.

Sensor	Major Technique Index		
IMU	Data rate: 200 Hz		
	Gyroscope	In-run bias stability	0.01 deg/h
		Scale factor	10 ppm
	Accelerometer	In-run bias stability	10 µg
		Scale factor	10 ppm
GNSS	GPS + GLONASS, dual frequency		
	Sampling rate: 1 Hz		
	Position accuracy: 2 cm + 1 ppm (RMS) in RT-2 LITE mode		

**Table 3:** Summary of the relative accuracy level in shortwave track irregularity measurement.

Integration Mode	Evaluation Method	Relative Accuracy (mm)		Threshold Values (mm)
		Vertical	Lateral	
GNSS/INS mode	MSE	0.82	0.92	0.67
	ADEV	0.52	0.58	0.16
Motion-constrained GNSS/INS mode	MSE	0.31	0.51	0.67
	ADEV	0.13	0.25	0.16
